# A pentatricopeptide repeat protein restores *nap* cytoplasmic male sterility in *Brassica napus*

**DOI:** 10.1093/jxb/erx239

**Published:** 2017-07-20

**Authors:** Zhi Liu, Faming Dong, Xiang Wang, Tao Wang, Rui Su, Dengfeng Hong, Guangsheng Yang

**Affiliations:** 1 National Key Lab of Crop Genetic Improvement, Huazhong Agricultural University, Wuhan, PR China; 2 National Research Center of Rapeseed Engineering and Technology, National Rapeseed Genetic Improvement Center (Wuhan Branch), Huazhong Agricultural University, Wuhan, PR China; 3 College of Plant Science and Technology, Huazhong Agricultural University, Wuhan, PR China

**Keywords:** *Brassica napus*, cytoplasmic male sterility, map-based cloning, mitochondria, pentatricopeptide repeat protein, restorer of fertility (*Rf*)

## Abstract

Two forms of male-sterile cytoplasm, designated *nap* and *pol*, are found in the oilseed rape species, *Brassica napus*. The *nap* cytoplasm is observed in most *B. napus* varieties, and it confers male sterility on a limited number of cultivars that lack the corresponding restorer gene, *Rfn*. In the present study, using linkage analysis in combination with 5652 BC_1_ progeny derived from a cross between a *nap* cytoplasmic male sterility (CMS) line 181A and a restorer line H5, we delimited the *Rfn* gene to a 10.5 kb region on chromosome A09, which contained three putative ORFs. Complementation by transformation rescue revealed that the introduction of *ORF2*, which encodes a pentatricopeptide repeat (PPR) protein, resulted in the recovery of fertility of *nap* CMS plants. Expression analysis suggested that the *Rfn* was highly expressed in flower buds and it was preferentially expressed in the tapetum and meiocytes during anther development. Further RNA gel blots and immunodetection suggested that the *Rfn* gene may play a complicated role in restoring the *nap* CMS. Our work laid the foundation for dissecting the molecular basis of CMS fertility restoration and the nuclear–mitochondrial interactions in CMS/*Rf* systems.

## Teaser

Map-based cloning provides evidence that the restorer gene *Rfn* for *nap* CMS in *Brassica napus* encodes a pentatricopeptide repeat protein and shares many properties with previously cloned restorer genes.

## Introduction

Cytoplasmic male sterility (CMS), a maternally inherited trait that fails to produce functional pollen, has been observed in >150 higher plant species. At the molecular level, CMS is typically associated with an aberrant chimeric gene in the mitochondria (Hanson and Bentolila, 2004). In most cases, the male sterility phenotype can be recovered by dominant nuclear genes, termed restorer of fertility (*Rf*) genes, which can specifically reduce the accumulation of CMS-associated RNAs or proteins in F_1_ hybrids. Thus, CMS/*Rf* systems provide both an ideal model for investigating nuclear–cytoplasmic interaction and a practical tool for facilitating hybrid seed production.

In oilseed rape species, *Brassica napus*, there are two different endogenous male-sterile cytoplasms, *nap* and *pol*. The *nap* cytoplasm is the most prevalent cytoplasm in *B. napus* germplasm, but it confers male sterility only in a limited number of cultivars that lack the corresponding fertility restorer gene, *Rfn*. In the absence of the nuclear restorer gene *Rfn*, plants carrying the *nap* cytoplasm display male sterility when grown under relatively low temperature (22/16 °C; 16/8 h), while the sterility can be gradually repaired or even completely reversed with an increase of temperature (Fan and Stefansson, 1986). Because flowering occurs in spring or summer and the environmental temperature gradually goes up, this specific temperature-sensitive male sterility is hardly used for hybrid production. In contrast, the *pol* CMS is highly stable in most cases; thus it can be widely used for commercial hybrid seed production (Fan and Stefansson, 1986; Zhou and Fu, 2007).

Previous studies indicated that *pol* CMS is associated with expression of the novel ORF, *orf224*, which is located upstream of a normal mitochondrial gene, *atp6* (Singh and Brown, 1991, 1993), while *nap* CMS is associated with a different but related ORF, *orf222*, that is co-transcribed with an exon of a *trans*-spliced gene, *nad5c*, and another short ORF, *orf139* (L’Homme *et al.*, 1997). The nuclear fertility restorer genes for *nap* and *pol* CMS, *Rfn* and *Rfp*, represent different alleles or haplotypes of a single nuclear locus (Li *et al.*, 1998). For both the *nap* and *pol* CMS systems, the nuclear restorer genes may take the role of down-regulating the expression of their corresponding mitochondrial CMS-associated transcripts (Singh and Brown, 1991; L’Homme *et al.*, 1997; Liu *et al.*, 2016). To validate the allelism of *Rfn* and *Rfp* and better understand how *Rfn* regulates CMS-associated transcripts, it is necessary to clone *Rfn*.

To date, except for *Rf2* (encoding a mitochondrial aldehyde dehydrogenase) for T-CMS in maize (Cui *et al.*, 1996), *Rf17* (encoding an acyl-carrier protein synthase-like protein) for CW-CMS in rice (Fujii and Toriyama, 2009), *Rf2* (encoding a mitochondrial glycine-rich protein) for LD-CMS in rice (Itabashi *et al.*, 2011), and *Rf1* (encoding a putative peptidase of the M48 family) for Owen-CMS in sugar beet (Matsuhira *et al.*, 2012), the rest of the cloned *Rf* genes all encode pentatricopeptide repeat (PPR) proteins (Bentolila *et al.*, 2002; Brown *et al.*, 2003; Kazama and Toriyama, 2003; Koizuka *et al.*, 2003; Komori *et al.*, 2004; Klein *et al.*, 2005; Wang *et al.*, 2006, 2013; Hu *et al.*, 2012; Tang *et al.*, 2014; Huang *et al.*, 2015; Igarashi *et al.*, 2016; Liu *et al.*, 2016). Such proteins are characterized by the succession of tandem degenerate motifs of ~35 amino acids. Based on the structure of the PPR motifs, PPR proteins can be divided into P and PLS subfamilies. P-type PPR proteins contain only canonical 35 amino acid repeats (P), whereas PLS PPR proteins consist of sequential repeats of P, long (L), and short (S) PPR motifs (Lurin *et al.*, 2004). P-type PPR proteins were shown to participate in multiple aspects of organelle RNA processing steps such as cleavage, splicing, stabilization, and/or translation of their target transcripts, while PLS PPR proteins have been almost exclusively associated with C-to-U RNA editing (Barkan and Small, 2014). It has been verified that PPR proteins have the ability to recognize ssRNA following a one-motif to one-base rule (Barkan *et al.*, 2012; Okuda *et al.*, 2014). Recent computational, biochemical, and structural studies indicated that the PPR motif first produces an antiparallel helix–turn–helix fold whose repetition then forms a solenoid-like structure (Barkan *et al.*, 2012; Yagi *et al.*, 2013; Yin *et al.*, 2013). Within each PPR repeat, the combinations of two amino acids, known as the ‘PPR code’ at two key positions (the 5th and the 35th), confer recognized RNA specificity (Barkan *et al.*, 2012;Ban *et al.*, 2013; Ke *et al.*, 2013; Yagi *et al.*, 2013; Yin *et al.*, 2013; Gully *et al.*, 2015; Shen *et al.*, 2016).

In the present study, we finely mapped and cloned the nuclear restorer gene *Rfn* for *nap* CMS in *B. napus* by using a map-based cloning strategy and preliminarily investigated how it regulates the fertility of *nap* CMS. The *Rfn* encodes a mitochondria-targeted PPR protein, and it may play a much more complicated role in restoring the *nap* CMS.

## Materials and methods

### Genetic analysis of *nap* CMS restoration

A *nap* CMS male-sterile line 181A, a male-fertile line H5, and three *pol* CMS lines (1141A, 245A, and 7492A) were used in this study. The cross between 181A as female parent and H5 as male parent yielded male-fertile F_1_ plants. Self-pollination of F_1_ plants yielded an F_2_ segregating population, which was used to analyze the genetic control of *nap* CMS restoration. The *pol* CMS lines 1141A, 245A, and 7492A as female were also each used to cross with H5 for genetic analysis. The individual fertility of each cross combination and self-pollination was assessed as previously described (Liu *et al.*, 2012) during the early flowering period.

### Fine mapping of the *Rfn* gene

The *nap* CMS line 181A as female was crossed with H5 to create the F_1_ plants, and the CMS plants were backcrossed with F_1_ plants as pollen donor to produce a backcross segregation population containing 5652 individuals. Polymorphic markers around the *Rfn* locus were developed based on the synteny region of *B. napus* and other *Brassica* species, which were then used for fine mapping of the *Rfn* gene.

### BAC screening, sequencing, and candidate gene prediction

A bacterial artificial chromosome (BAC) clone library described by Li *et al.* (2015) was screened with co-segregated markers through a two-stage PCR screening method (Crooijmans *et al.*, 2000). The target BAC clone was then sequenced by Illumina Hiseq 2000 according to the manufacture’s standard protocol. The online-based tool FGENESH (http://www.softberry.com) was used to predict putative ORFs in the delimited gene region. The genomic or coding sequences (CDSs) of predicted genes were submitted to BRAD (http://brassicadb.org;Cheng *et al.*, 2011) for homology search and basic functional annotation.

### Complementation by transformation rescue

The genomic sequence of candidate gene (including the putative promoter, CDS, and 3'-downstream region) was PCR amplified with H5 as a template using specific primers (see Supplementary Table S1 at *JXB* online). The PCR product was inserted into the *Eco*RI and *Bam*HI sites of the binary vector pFGC5941 and then introduced into the CMS line 181A by *Agrobacterium*-mediated transformation. Individual transgenic plants were raised to maturity and visually assessed for male fertility/sterility under relatively low temperature during flowering time. Self-pollination of the fertility-recovered T_0_ plants produced T_1_ plants, and the CMS parent line as female was backcrossed with T_0_ plants to produce test cross progeny plants. Pollen grains from the anthers of the transgenic plants were collected before anthesis and dyed in 1% aceto-carmine staining solution. The stained pollen was photographed under a microscope.

### RNA extraction and quantitative reverse transcription–PCR

Total RNA was extracted from various rapeseed tissues using an RNeasy plant mini kit (Qiagen, http://www.qiagen.com/). RNA samples of ~2 μg were converted into cDNA with GoScript™ Reverse Transcriptase following the manufacturer’s instructions (Promega, USA), and the products were diluted 10-fold with distilled water for template. Quantitative reverse transcription–PCR (qRT–PCR) was performed with the Bio-Rad CFX96 Real-time system (Bio-Rad) and the GoTaq qPCR Master Mix (Promega, USA). Relative expression levels were calculated by the 2^–ΔΔCt^ method (Livak and Schmittgen, 2001). The relative amount of PCR product that was amplified using the designed primer sets (listed in Supplementary Table S1) was normalized to the internal control gene *BnActin* (GenBank: AF111812.1). The data are expressed as the mean ±SD (*n*=3 biological replicates).

### GUS staining assay

An ~2000 bp upstream region of the *Rfn* gene was amplified from the restorer line H5 (Primers Pro-F and Pro-R; Supplementary Table S1) and inserted into the cloning vector pMDC162. The Pro_*Rfn*_–GUS (β-glucuronidase) construct was then introduced into wild-type Arabidopsis plants by floral-dip transformation (Clough and Bent, 1998). GUS activity was visualized by staining various tissues from the heterozygous transgenic plants and wild-type plants overnight at 37 °C in X-Gluc solution containing 50 mM sodium phosphate buffer of pH 7.0, 10 mM EDTA, 1 mM X-Gluc (5-bromo-4-chloro-3-indolyl-glucuronide), and 20% methanol (w/v). The stained samples were cleared in 75% (v/v) ethanol and then photographed under a stereomicroscope (Olympus SZX16, Japan).

### RNA *in situ* hybridization analysis

Floral buds at different developmental stages from the restorer line H5 were freshly collected and fixed in FAA (50% ethanol, 5.0% acetic acid, and 3.7% formaldehyde). Fixed floral buds were dehydrated with a 50–100% ethanol series, cleaned by a series of xylenes from 25% to 100%, and embedded in Paraplast. Anther sections (8 μm thickness) were cut using a Leica RM2235 microtome and placed on RNase-free polylysine-coated slides. A 124 bp CDS fragment from the *Rfn* gene was amplified using the insitu-F and insitu-R primers (Supplementary Table S1) and inserted into the pGEM-T Easy vector (Promega, USA) for sequencing. The probes were labeled with digoxigenin (DIG) using an RNA labeling kit (Roche Applied Science). The subsequent hybridization and immunological detection procedures were performed as previously described by Wan *et al.* (2010).

### Transient expression and subcellular localization of RFN

The coding sequence of the putative N-terminal 44 residue signal peptide was PCR amplified using primers *Rfn-Xba*-F and *Rfn-Xba*-R (Supplementary Table S1) and then inserted into the pM999-GFP vector to generate a green fluorescent protein (GFP) fusion product. The fusion construct was introduced into Arabidopsis protoplasts by polyethylene glycol (PEG)/calcium-mediated transformation (Yoo *et al.*, 2007). The mitochondria of protoplasts were marked using MitoTracker Red CMX-Ros (Molecular Probes, Invitrogen) staining solution after incubation at 23 °C for 14–18 h. GFP expression and co-localization of GFP fusion protein to mitochondria were imaged using a confocal scanning microscope system (Olympus FV1200, Japan) with 488 nm laser light for fluorescence excitation of GFP and 580 nm laser light for excitation of MitoTracker Red CMX-Ros.

### RNA gel blot analysis

For RNA gel blot analysis, total RNA was isolated from young flower buds using Trizol reagent (Invitrogen, http://www.invitrogen.com/) as described by the manufacturer. Approximately 20 μg of total RNA from each sample was size fractionated on 1.2% agarose/2.2 M formaldehyde horizontal gels and transferred to Hybond-N^+^ nylon membranes (Amersham) by capillary blotting with 3 M NaCl/0.3 M sodium citrate for 16–20 h. The *orf222* and *orf139* fragments were amplified from 181A using the primers listed in Supplementary Table S1. The PCR products were inserted into the pGEM-T Easy vector (Promega, USA) for sequencing. Antisense DIG-labeled RNA probes were made with the RNA labeling kit (Roche Applied Science). The hybridization and detection with DIG Easy Hyb and the DIG Nucleic Acid Detection Kit (Roche Applied Science) were performed following the manufacturer’s instructions.

### Antibody preparation and immunodetection

A peptide antigen (QSKLKLGGKDRTSK) corresponding to 14 residues of ORF222 was synthesized by a chemical synthesis method (GenScript, Nanjing, China) and used to immunize rabbits for antibody production. Total proteins were extracted from the young flower buds of CMS line, restorer line, and transgenic fertility-restored plants. The proteins were separated by 12% SDS–PAGE and transferred onto a membrane (PVDF type, Millipore). The following procedures were performed as previously described by Jing *et al.* (2012).

## Results

### Male fertility restoration is controlled by one dominant gene

To investigate the genetic control of fertility restoration of *nap* CMS, we prepared an F_2_ population derived from the cross of a *nap* CMS line 181A as female and a male fertile line H5 (Supplementary Fig. S1). Fertility assessment showed that 715 of 940 plants had a fertile phenotype and the remaining plants had a sterile phenotype, fitting the 3:1 segregation ratio (χ^2^=0.51, *P*<0.05) of a single locus, whereas all the F_1_ plants derived from the crosses between three *pol* CMS lines and H5 remained male sterile (Table 1). These results indicated that the male-fertile line H5 can specifically restore the *nap* CMS line 181A in a sporophytic manner and possesses only one dominant *Rfn* gene.

**Table 1.** Genetic analysis of the fertility restorer gene in H5The male-sterile lines 1141A, 245A, and 7492A are *pol* cytoplasm; the male-sterile line 181A is *nap* cytoplasm

Cross combination	Progeny	Total plants	Fertile plants	Sterile plants	Expected ratio	χ^2^ value^*a*^
1141A (*pol*)×H5	F_1_	115	0	115	–	–
245A (*pol*)×H5	F_1_	67	0	67	–	–
7492A (*pol*)×H5	F_1_	69	0	69	–	–
181A (*nap*)×H5	F_1_	83	83	0	–	–
	F_2_	940	715	225	3:1	0.51

^*a*^χ^2^ (0.05, 1)=3.84.

### The *Rfn* locus was limited to a 10.5 kb region

**Fig. 1.**
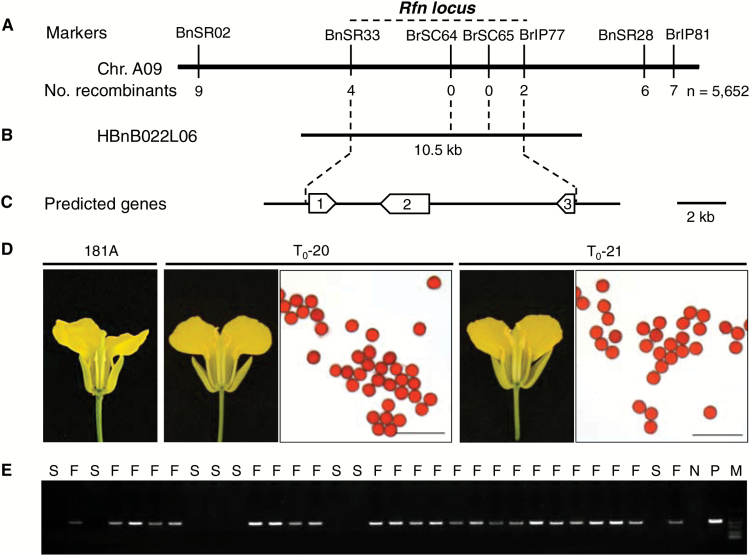
Map-based cloning of the *Rfn* gene for *nap* CMS in *Brassica napus*. (A) Molecular mapping of the *Rfn* locus using a BC_1_ population containing 5652 individuals. The numbers of recombinants between the markers and *Rfn* are shown. (B) Physical map based on a sequenced BAC. The *Rfn* locus was narrowed down to a 10.5 kb region. (C) The predicted genes from http://www.softberry.com. It contained two complete CDSs (*ORF1* and *ORF2*) and a partial CDS (*ORF3*). (D) Morphology of flowers and pollen grains of a *nap* CMS plant and two hemizygous *Rfn* candidate transgenic plants. Dissected flowers were photographed with a digital camera. Pollen grains were stained with 1% aceto-carmine staining solution. Scale bar=100 μm. (E) Co-segregation analysis of the introduced DNA and fertile plants among the T_1_-20 progeny. F, fertile plant; S, sterile plant; N, negative control; P, positive control; M, DL2000 molecular markers. (This figure is available in colour at *JXB* online.)

A previous study indicated that the *Rfn* and *Rfp* genes represent different alleles or haplotypes of a single nuclear locus (Li *et al.*, 1998). To map the *Rfn* locus precisely, sequence information from the collinear region around the *Rfp/Rfn* locus was used for development of simple sequence repeat (SSR) or sequence characterized amplified region (SCAR) markers. Subsequently, 16 markers tightly linked to the *Rfn* gene were successfully obtained. These markers were further used to assay each individual of the backcross segregation population. Among these plants, four and two recombinant individuals were identified using the closest flanking markers BnSR33 and BrIP77, respectively, and two markers (BrSC64 and BrSC65) co-segregated with the *Rfn* locus (Fig. 1A). To characterize precisely the genomic sequence around the *Rfn* locus in *B. napus*, we further screened a BAC clone library using the two co-segregated markers; a BAC clone (HBnB022L06) was also successfully identified. After sequencing the target BAC clone and sequence alignment of the closest flanking markers, the *Rfn* locus was narrowed down to a 10.5 kb physical region on chromosome A09 of *B. napus* (Fig. 1B). Gene prediction showed that this region contained two complete CDSs (*ORF1* and *ORF2*) and a truncated CDS (*ORF3*) (Fig. 1C). Homologous gene annotation in Arabidopsis showed that *ORF1* and *ORF2* encode a zinc finger (AN1-like) family protein and a PPR-containing protein, respectively, while *ORF3* encodes a serine-type endopeptidase. Considering most *Rf* genes encoding PPR proteins, we took *ORF2* as the *Rfn* candidate.

### Validation of the target gene of *Rfn* for *nap* CMS

To validate *ORF2* as the target of *Rfn*, we isolated the genomic sequence (including the putative promoter, ORF, and 3'-downstream region) of *ORF2* from the restorer line H5 and cloned it into binary vector pFGC5941. The construct was then introduced into the *nap* CMS line 181A by *Agrobacterium*-mediated transformation. Nine independent T_0_ transgenic plants displayed a normal male fertile phenotype when grown to maturity under relatively low temperature (Fig. 1D). The recovered fertility of T_1_ progeny plants could be stably co-transmitted and co-segregated with the introduced DNA (Fig. 1E). Meanwhile, a 3:1 or 15:1 genetic segregation ratio was observed in the five independent transgenic T_1_ progeny of the self-cross and a 1:1 or 3:1 genetic segregation ratio was observed in the corresponding test-crossed progeny (Supplementary Table S2). These results suggested that a single site or two independently heritable T-DNA insertions was introduced into 181A in the T_0_ transgenic plants. Thus, our data proved that *ORF2* possesses the ability to restore the *nap* CMS in a sporophytic manner and it is the causal gene of *Rfn*.

### Sequence analysis of the *Rfn* gene and its recessive allele

**Fig. 2.**
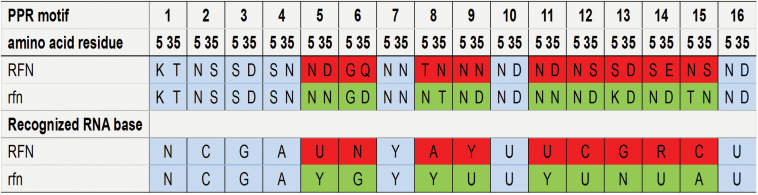
Combinations of amino acid residues at positions 5 and 35 of PPR motifs and the RNA sequence predicted to be recognized by these combinations for RFN and rfn proteins. ‘N’ represents that the recognized RNA base is unknown; ‘R’ represents that the recognized RNA base is A or G; ‘Y’ represents that the recognized RNA base is U or C. The same or different amino acid residue combinations in each PPR motif between RFN and rfn and their recognized RNA bases are indicated by different shading. (This figure is available in colour at *JXB* online.)

To characterize the recessive allele of *Rfn* existing in the CMS line, *ORF2* was amplified from the 181A genome and sequenced. Comparing the putative promoter region (~2.0 kb) and the full-length CDS of *Rfn* from H5 with that of the *rfn* allele from 181A, we found that only five single nucleotide polymorphisms (SNPs) were detected in the putative promoter region, while rich sequence variations including 184 SNPs were identified in the coding sequence. These SNPs have no effect on the deduced protein length, but result in 99 amino acids mutations (Supplementary Figs S2, S3). Sequence analysis indicated that both *Rfn* and *rfn* encode novel PPR proteins (RFN and rfn, respectively) of 629 amino acids with 16 PPR motifs (Supplementary Fig. S4), which contain a predicted mitochondrial transit signal at the N-terminus (https://ihg.gsf.de/ihg/mitoprot.html;Claros and Vincens, 1996). Further analysis of the PPR motifs between RFN and rfn indicated that nine of the ‘code’ combinations in each PPR motif are different from each other (Fig. 2). This difference suggested that the recognized sequence or the binding ability of CMS-associated RNA target sequence may be different, and thus cause the functional divergence between RFN and rfn.

### Expression pattern of the *Rfn* gene

**Fig. 3.**
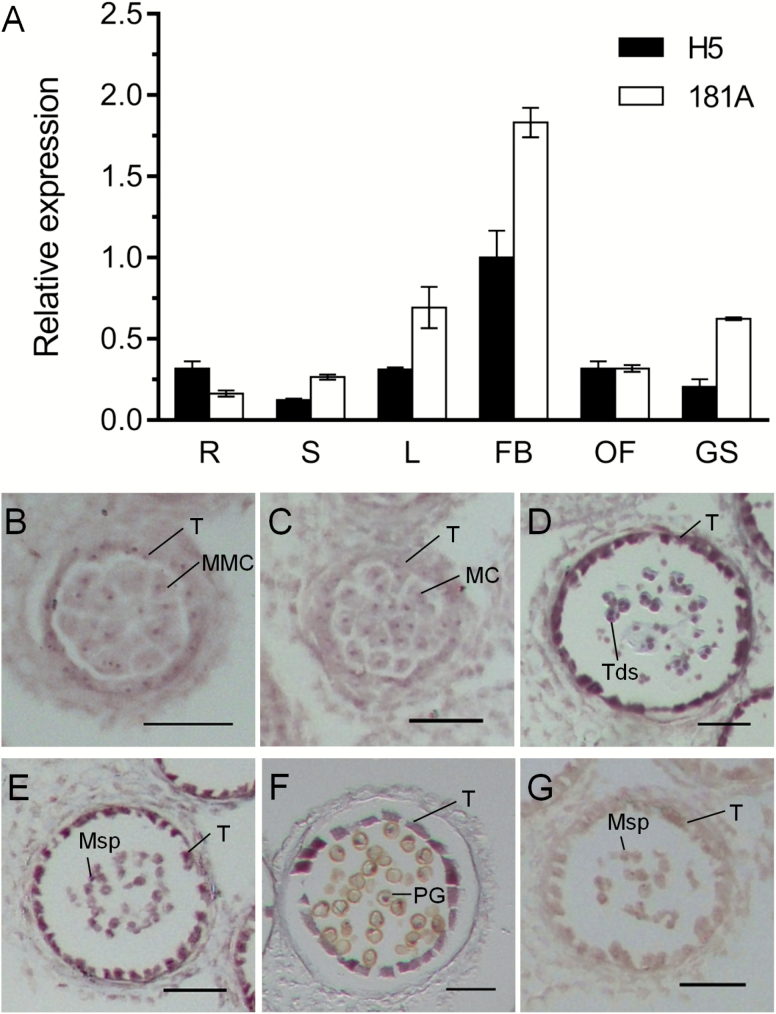
The expression patterns of *Rfn* (*rfn*) by qRT–PCR and RNA *in situ* hybridization. (A) qRT–PCR analysis of *Rfn* and *rfn* expression in the selective tissues of parent lines H5 and 181A. R, root; S, stem; L, leaves; FB, flower buds; OF, opening flowers; GS, green siliques. The expression levels were normalized to *BnActin*. The values are presented as the means ±SD (*n*=3 biological replicates). (B–G) The expression patterns of *Rfn* by RNA *in situ* hybridization in the anther of the fertile parent H5 at different developmental stages detected by the antisense probe (B–F) and sense probe (G). MC, meiotic cell; MMC, microspore mother cell; Msp, microspores; PG, pollen grains; T, tapetum; Tds, tetrads. Scale bars=50 μm. (This figure is available in colour at *JXB* online.)

**Fig. 4.**
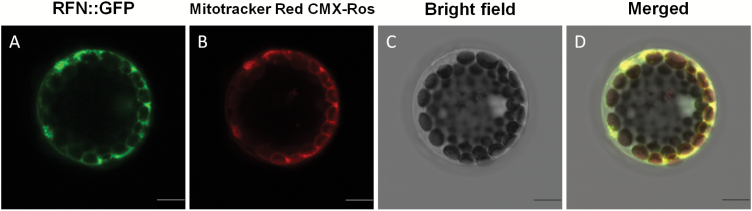
Subcellular localization of the N-terminal 44 residue signal peptide of RFN fused with green fluorescent protein (GFP). (A) The protoplast showed a green fluorescent signal at 488 nm. (B) The same protoplast showed a red fluorescent signal (stained by MitoTracker Red CMX-Ros) at 580 nm. (C) The bright-field image. (D) The merged image of (A), (B), and (C). Scale bars=10 µm. (This figure is available in colour at *JXB* online.)

qRT–PCR analysis showed that the *Rfn* and *rfn* alleles are constitutively expressed in the selected tissues, and both maintained relatively high-level expression in the flower buds (Fig. 3A). Similar results were obtained by GUS staining assay in the *Rfn* promoter–GUS fusion transgenic Arabidopsis plants (Supplementary Fig. S5). To determine precisely the spatial and temporal expression pattern of *Rfn* during different anther development stages, we performed RNA *in situ* hybridization using an *Rfn*-derived probe. As shown in Fig. 3, faint hybridization signals were visible at the microspore mother cell (MMC) and meiotic stages (Fig. 3B, C). At the tetrad stage, the expression of the *Rfn* gene was detected in the tapetum and tetrads at its maximal level (Fig. 3D). The hybridization signals were slightly weakened in the subsequent stages and remained detectable in the tapetum but sharply decreased in the pollen grains (Fig. 3E, F). Subcellular localization analysis using a construct expressing a GFP fused with the N-terminal 44 residue signal peptide of RFN confirmed that this protein targets to mitochondria (Fig. 4), consistent with its function in restoring CMS.

### Effects of Rfn on processing of *nap* CMS-associated transcripts or protein

**Fig. 5.**
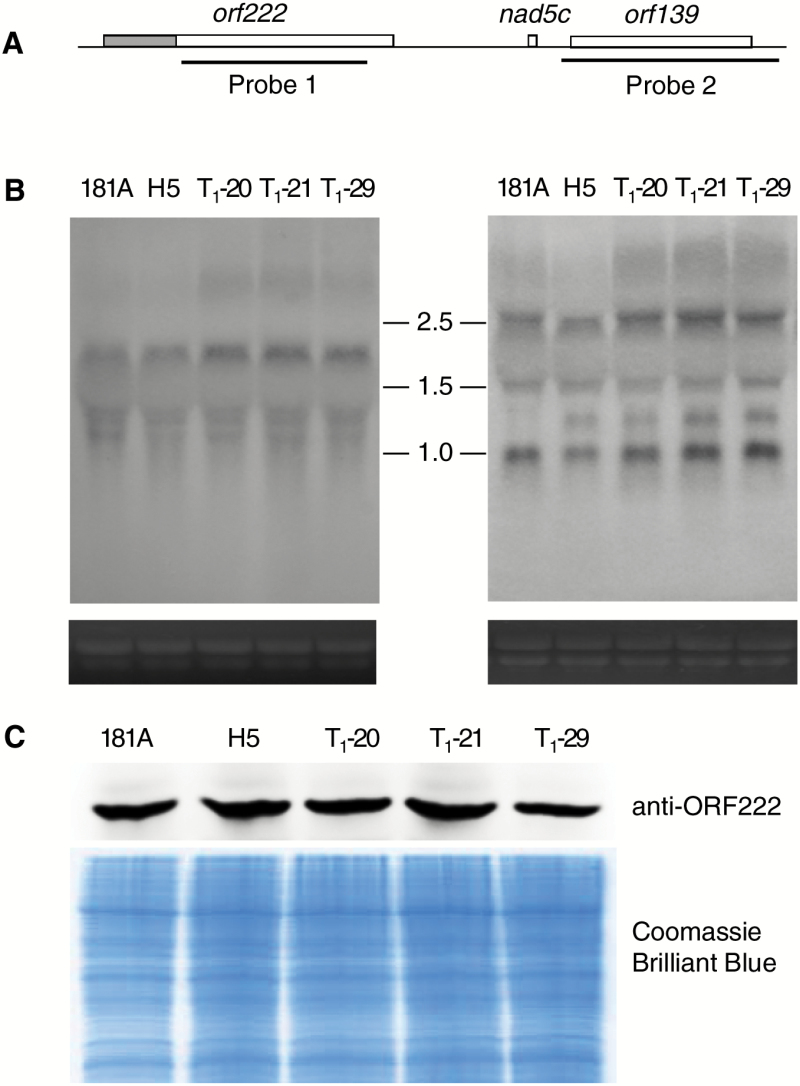
RNA gel blot analysis of the *orf222/nad5c/orf139* transcripts with probes *orf222* and *orf139*, respectively, and immunodetection of ORF222 from CMS, restorer line, and transgenic fertility-restored plants. (A) The structure of *nap* CMS-associated transcripts *orf222/nad5c/orf139*. The shaded portion indicated the *orfB* homologous region. (B) RNA gel blots analysis with probes *orf222* (left panel) and *orf139* (right panel), respectively. The estimated sizes of the different transcripts are shown in kilobases. Ethidium bromide stain of the gel confirms equal RNA loading. (C) Immunodetection of CMS protein expression in T_1_ progeny with an anti-ORF222 antibody. Equal amounts of total protein were loaded in each lane stained by Coomassie Brilliant Blue. (This figure is available in colour at *JXB* online.)

In a previous study, northern blot analysis suggested that expression of the *orf222/nad5c/orf139* region may be associated with the *nap* CMS (L’Homme *et al.*, 1997). To examine the mechanism through which RFN affects this CMS-associated transcript, RNA gel blot analysis was performed with the probes *orf222* and *orf139*, respectively. As shown in Fig. 5, the *orf222*-containing transcript levels in the restorer and transgenic fertility-restored lines were not significantly changed when compared with those in the *nap* CMS line (Fig. 5B). In contrast, an ~1.2 kb transcript was specifically observed in the restorer and transgenic fertility-restored lines when using *orf139* as a probe (Fig. 5B). Further immunodetection by the antibody against the *orf222*-encoded protein (ORF222) showed that the accumulation of ORF222 was not obviously changed in the restorer line and transgenic fertility-restored lines compared with the CMS line (Fig. 5C). These results indicate that the *Rfn* gene may not responsible for processing the *orf222* transcripts but rather the *orf139* transcripts.

## Discussion

### *Rfn* and *Rfp* are tightly linked independent genes and highly homologous

A previous study indicated that the restorer genes *Rfn* for *nap* cytoplasm and *Rfp* for *pol* cytoplasm represent different alleles or haplotypes of a single nuclear genetic locus (Li *et al.*, 1998). Very recent research confirmed that *Rfn* indeed localizes to the same genomic region as *Rfp* (Gaborieau and Brown, 2016). In this study, through a map-based cloning strategy, we finely mapped the *Rfn* gene on the end of chromosome A09 of *B. napus* and successfully isolated it; and it was shown to encode a novel mitochondria-localized PPR protein. According to the *B. napus* reference genome of Darmor-*bzh* (Chalhoub *et al.*, 2014), we found that the physical distance between of *Rfn* and *Rfp* (Liu *et al.*, 2016) is ~125 kb. Sequence comparison showed that RFN and RFP are highly conserved, due to their sequence identity of 70% at the amino acid level (Supplementary Fig. S4). BlastP showed that both RFN and RFP have the highest similarity to RFL2 (At1g12300) in *Arabidopsis thaliana* (Supplementary Fig. S4). However, the gene *Rfp* which can restore the *pol* CMS does not possess the function in recovering the *nap* CMS, because some *pol* CMS restorer lines failed to restore the *nap* CMS of 181A in F_1_ plants, and all the positive T_0_ transformants remained male sterile phenotype when we introduced *Rfp* into 181A (data not shown). So, our results strongly suggested that *Rfn* and *Rfp* are tightly linked independent genes and they are likely to be evolutionarily homologous genes, but underwent a diversifying selection for adapting to newly emerging sterility-inducing genes (Geddy and Brown, 2007).

### The possibility of functional divergence between RFN and rfn

PPR proteins constitute a large group of RNA-binding proteins in terrestrial plants, and function as sequence-specific ssRNA-binding proteins mainly in chloroplasts and mitochondria, where they are involved in various steps of organelle gene expression (Barkan and Small, 2014). Recent structural studies of PPR proteins indicated that one PPR motif has the ability to recognize a single nucleotide, and the amino acid combinations at the 5th and 35th residues of each PPR motif are very important for recognizing the specific RNA sequence (Yin *et al.*, 2013; Shen *et al.*, 2016). In *A. thaliana*, an amino acid mutation at position 35 of the third PPR motif results in RFL9 losing its function of processing the *rps3* and *orf240* transcript in the mitochondria (Arnal *et al.*, 2014). The comparison of coded amino acids combinations between RFN and rfn showed that nine of them are different. Furthermore, we found that *Rfn* and *rfn* are constitutively expressed in various tissues, and the expression level of *rfn* is even higher than that of *Rfn* in flower buds. So, it is suggested that the functional divergence between RFN and rfn may not due to their few sequence variations in promoter regions, but have resulted from the changes in nine amino acids residing in the code positions.

### The possible role of Rfn

The restoration of fertility in CMS/*Rf* systems may be achieved by various mechanisms at different molecular levels, such as the genomic level, post-transcriptional level, translational or post-translational level, and metabolic level (Chen and Liu, 2014). Most CMS-associated transcripts or proteins in various CMS crops are cleaved or degraded by the corresponding RF proteins. In BT-CMS rice, the CMS-associated transcripts *B-atp6/orf79* were cleaved by RF1A but degraded by RF1B (Wang *et al.*, 2006). In Ogu-CMS rapeseed, the RFO protein may impede *orf138* mRNA translation by preventing either association or progression of mitochondrial ribosomes with the *orf138* mRNA (Uyttewaal *et al.*, 2008). A previous study indicated that the *nap* CMS-associated transcripts *orf222/nad5c/orf139* were affected by the action of *Rfn* at the post-transcriptional level (L’Homme *et al.*, 1997). In the current work, we detected the CMS-associated transcripts in the *nap* CMS line, restorer line, and transgenic fertility-restored lines using the *orf222* and *orf139* probe, respectively. We found that the CMS-associated transcripts were not significantly changed when using *orf222* as a probe, but an ~1.2 kb transcript appeared in the restorer line and transgenic fertility-restored lines when *orf139* was used as a probe. The accumulation of ORF222 was also not obviously changed in the restorer line and transgenic fertility-restored lines compared with the *nap* CMS line. These findings suggested two possibilities: first, there is another CMS-associated gene but not *orf222*; secondly, RFN may process the *nap* CMS-associated *orf222* in a unique stage. Further investigation is needed to identify the target RNA of RFN or RNA blotting with a specific stage of the anther and thus illustrate the molecular mechanism of how RFN recovers the *nap* CMS in *B. napus*.

## Supplementary data

Supplementary data are available at *JXB* online.

Fig. S1. Opening flower morphology of the parent plants 181A and H5 under relatively low temperature.

Fig. S2. The putative promoter region and coding sequence of *Rfn* and its deduced amino acids from the restorer line H5.

Fig. S3. Nucleotide sequence of recessive *rfn* allele from 181A and the deduced amino acids.

Fig. S4. Alignment of the amino acid sequences of RFN, RFP, and their homologous protein RFL2 in Arabidopsis.

Fig. S5. GUS assay from different tissues of Pro_*Rfn*_:GUS-introduced Arabidopsis plants.

Table S1. The primers used in this study.

Table S2. Fertility of T_1_ progeny derived from five independent T_0_ transgenic plants by selfing and test cross.

## Supplementary Material

Supplementary_Figures_S1_S5_Table_S1_S2

## Abbreviations:

BAC: bacterial artificial chromosome
CDS: coding sequence
CMS: cytoplasmic male sterility
DIG: digoxigenin
GFP: green fluorescent protein
PPR: pentatricopeptide repeat
qRT–PCR: quantitative reverse transcription–PCR
Rf: restorer of fertility.
