# Supplementary material for: A pentatricopeptide repeat protein restores nap cytoplasmic male sterility in Brassica napus
Source: J Exp Bot. 2017 Jul 20;68(15):4115–23. doi: 10.1093/jxb/erx239 (PMC5853434; doi:10.1093/jxb/erx239)
Supplement: Supplementary_Figures_S1_S5_Table_S1_S2 [file erx239_supplementary_figures_s1_s5_table_s1_s2.pdf]

Supplementary data

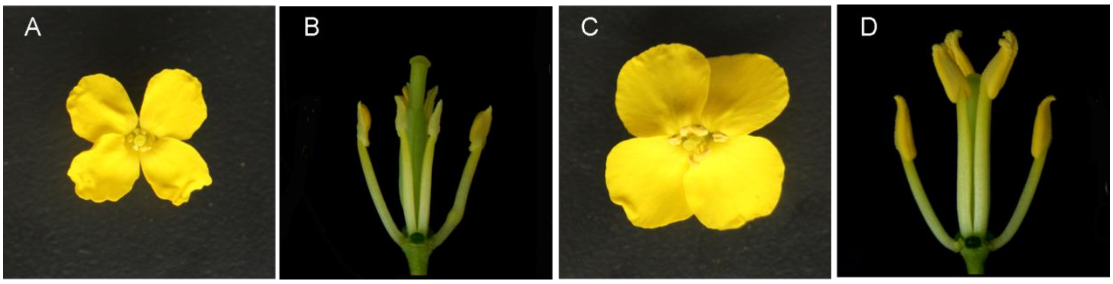

Supplementary Fig. S1. Opening flower morphology of the parent plants 181A and H5 under relatively low temperature. A, the whole flowers of sterile parent 181A; B, the sterile flower with petals and sepals peeled off; C, the whole flowers of fertile parent H5; D, the fertile flower with petals and sepals peeled off. The petals of sterile parent are shrinked (A) and the stamens of sterile parent are shorter than pistil (B).

```
-1980  CTGGTACATACAGTATCAGTGTTAATGATGTCGTTCTAAAAACATTTGATGTTCATCAGAACATACCTTTCACAATCGAAATCATTGATG
-1890  ATAACCAGGTTTGTCTTTTTGTTCTGTGCGTGTATATTTTACTCTCTTTATTGAACTAGTCAATCATTTGTTAATATGCTTTCACAGATT
-1800  CCTCTTGCTGGTGAAAAATTAATTTTTGATAAGTCAGTGTCTATAGTCACTGATGGGGGACATGTTGTTTCTAAGGGTAAGAAAAATTGA
-1710  ACAACGATTTAGAATTTGTAATCAATTCTCAGTTCTCAATAGACTAATTACAACAACCTTTTGGTGCAGGTGTCATTAAGCTGCCATTTT
-1620  TAGGGAGAACGGACTAGATGCCATAGAGGTAAAAAGCTTACACGTAATCCAAGAGGTCTCCACAGATGTTAACAACCAGAAGAAATAATA
-1530  ATAATGTTTTGCCGTACTGGTTCAGTGTTTTTTGTTTCTGTAATGACTATCAACAAAACTTTTGTTAGACAAGCAATGAAAACGCAACGC
-1440  AGGTGAGGAGAGGCCATATATGGATCATAAGCTTAATTTCAAATGAATGATTTATGCAAGTGTTTTAATGTATTAGTCTTCGTTGTATAA
-1350  GAATATTTATCAAAAAAATTTGAAGTAGACTCATGTTTTGTTCCAGTAAGTTTTCTCTTAAACCAACTAAATATTTGTGTACAAAATTTA
-1260  CACGGCCAAAGAAAGATCAACTCAATACATAATTTAAGTTTGTATGGAAACAAACATTCATGCTTCAAGATACATAAATAAGCACGTTTA
-1170  TGTGTGTATATCAGTGAAATTGTCTTTTTCTATCGATGATCTTTTATCTCTCCTTCAATAGCTTTAGGAGATAAAGATGCATGATCATAA
-1080  TGGTCCACATATTAAAAAAAAACACAGTAAATATAAAATCCATAAGGCATTAAATAAAACATTGGTTAAAGTTCCTATAAACTCTCGAAC
-990   TCAGTTTCTTACAACTTCAAGTCTCCATTTCTGTAGCACTTTCTTTGTGAACTTTACTGAATTGTGGACCACAAAAGTCACACTCCTTAT
-900   ATATGAAAATGGCTTAAGATTGCTTTTTGGATTTTTGCTATGTTGATTACAATCGTTATCATGACGCTACCATGATTGGATAGTAAGAAC
-810   AAAATTTAAGTGTATTTCTTTAAGTGAAGTTAAAAAGATACAAATCACTCATTTTGCAACTCTAAAAGTTATATCAAGAATCTCTACCAA
-720   AATTTCTAAAGCATACTTGAAACCAGGATATTCTTTTGTGTTTCACAATATGATTAACACCGTGAGTTGCATTGAATTTCTTTAGCAAAT
-630   AGATATGTAGGGGTAATGATGAATTTGAATTAGCGTTGTTTCTATGAAGCTTTAAGCAAACCGGAAATGATGGGGGTTAATAACCAAGAA
-540   TGAGCATGAACCGGCCTTGAGGTGTGAATGATTTGATTGATCCCAATATAGTGATATTTAGTTCCTTAATGGAGCTGTCAACAAATTATA
-450   AACTGAAATTGTGTAAAACAGAACTAAAATGAAATCGGACATGTTACTACGACATATCTCCTGTAATTATTAACATTGAAAATGAAATAA
-360   ATAAATAAAAAAAAAAGAAAAAAAGAAATCGGACATGATATAACGCGGTCTGCTTTTATACAAACCAGATTCGTACCGCTTCTGAAACCA
-270   AATTAGATTAATTTCAGAACCGAGACGTGATTTAAAAAAAAAAAAAACAGATACAACACACTTTCCCTTGTCTTCTTCGTCGTGTTCTTGA
-180   CCGGGATCTACAAAGTTAACGATGGAATGTTTCTGCTGTAGATAAAATTCGTCTGGTGGTGAAGATGTTTTTGTGGTGAAAGGCGAAGAA
-90    TCATCAAAGTCAGTGAAGACGGGAGTGGAGTTGTTCTTCTTCTTCTAGGGGTTTTCCAGATTTGTAGATTTGAAGAAAAAAAAACAAACA

1      atgttgtgtcggagattggtgcttgtgtctcgtatccccctgagtcctgttggtactcttgcaactcctttgctctcttttatcagatcc
       M  L  C  R  R  L  V  L  V  S  R  I  P  L  S  P  V  G  T  L  A  T  P  L  L  S  F  I  R  S
91     tcatgcgaacgaggctactctggtctcggcagcgatagaaatctctcatcttacaaagagagactgagaagtggtctggtcgatatcaag
       S  C  E  R  G  Y  S  G  L  G  S  D  R  N  L  S  S  Y  K  E  R  L  R  S  G  L  V  D  I  K
181    aagaaggatgctgtagctctgtttcagtccatgattaggtctcgtcctcttcctacggtcatggatttcaataaactgtttagtgcagta
       K  K  D  A  V  A  L  F  Q  S  M  I  R  S  R  P  L  P  T  V  M  D  F  N  K  L  F  S  A  V
271    gccagaacgaaacagtatgatctcgtgttggatctctgcaagcaaatggaactgcaagggattgcacatagcatttacacgctgagtatt
       A  R  T  K  Q  Y  D  L  V  L  D  L  C  K  Q  M  E  L  Q  G  I  A  H  S  I  Y  T  L  S  I
361    atgatcaattgcttctgccgcctccagaaactcggttttgctttttctgtgatgggaaagatgttgaagcttgggtatgagcccgacaca
       M  I  N  C  F  C  R  L  Q  K  L  G  F  A  F  S  V  M  G  K  M  L  K  L  G  Y  E  P  D  T
451    atcacattctcaactttgatcaacggtttatgtctggtgggtagagtttccaaagctgtggagttagttgatcatatggtagatatgaag
       I  T  F  S  T  L  I  N  G  L  C  L  V  G  R  V  S  K  A  V  E  L  V  D  H  M  V  D  M  K
541    gttattccaaatctcatcatacttaacactattgtcaatgggctttgtctccaagatagactctctgaagcaatgtctttgatacatcga
       V  I  P  N  L  I  I  L  N  T  I  V  N  G  L  C  L  Q  D  R  L  S  E  A  M  S  L  I  H  R
631    atgttggctaatgggtgccaacccgacgcagttacatatggtccggttttgaacagaatgtgtaagtcagggaacactgcctcggccttg
       M  L  A  N  G  C  Q  P  D  A  V  T  Y  G  P  V  L  N  R  M  C  K  S  G  N  T  A  S  A  L
721    gatctcctcggaaagatggaacttagaaagatcaagcctcaagtagtcacatacaatatcatcattgacagtctttgcaaggatgggagc
       D  L  L  G  K  M  E  L  R  K  I  K  P  Q  V  V  T  Y  N  I  I  I  D  S  L  C  K  D  G  S
811    ctcgaagatgcactctgccttttcaatgaaatggaaaccaaaggaatcaaagcaaatgtcattacctacacctctctcataggaggcttt
       L  E  D  A  L  C  L  F  N  E  M  E  T  K  G  I  K  A  N  V  I  T  Y  T  S  L  I  G  G  F
901    tgtagtgccggaagatgggatgatggtgcacagttgctgagggatatgattacaaggggagtcacccctaacgttgtcactttcaatgct
       C  S  A  G  R  W  D  D  G  A  Q  L  L  R  D  M  I  T  R  G  V  T  P  N  V  V  T  F  N  A
991    ttgattgatagttttgtgaaagaaggacagcttaccgaggctaaaaaattgtacaatgagatgatcacaagaggcacagatccaaatatc
       L  I  D  S  F  V  K  E  G  Q  L  T  E  A  K  K  L  Y  N  E  M  I  T  R  G  T  D  P  N  I
1081   attacatataactctttgatatatgggatgtgcatggacaactgcctagatgaggccaaccagatgctggatatgatggttagcaaggga
       I  T  Y  N  S  L  I  Y  G  M  C  M  D  N  C  L  D  E  A  N  Q  M  L  D  M  M  V  S  K  G
1171   ttctatcctgatattgtgacgtttaatatccttatcaacggatactgtaaggctaaacaggtcgatgaaggtacgagactttccgcaat
       F  Y  P  D  I  V  T  F  N  I  L  I  N  G  Y  C  K  A  K  Q  V  D  E  G  T  R  L  F  R  N
1261   atgtgtttaagaggagtggttgctgatacagtcacttataacactctcatccaagggttttgtcaatcgggaaaacttaatgttgccaaa
       M  C  L  R  G  V  V  A  D  T  V  T  Y  N  T  L  I  Q  G  F  C  Q  S  G  K  L  N  V  A  K
1351   gaactcttccaggagatggtctttgttggtgtgccacccagtgttgtgacttatagtattttgctggatgggttgtgtgacaatggggaa
       E  L  F  Q  E  M  V  F  V  G  V  P  P  S  V  V  T  Y  S  I  L  L  D  G  L  C  D  N  G  E
1441   ctagaaaaggctttggaaatacttgatcaaatgctcaagagtaagatggaacttgacattggtatatatagtatcatcattcacgggatg
       L  E  K  A  L  E  I  L  D  Q  M  L  K  S  K  M  E  L  D  I  G  I  Y  S  I  I  I  H  G  M
1531   tgcaatgctagtaagatcgatgatgcttgggatctattctgtagcctccctctcaaaggagtgaagcctgaagtcagaacatataatata
       C  N  A  S  K  I  D  D  A  W  D  L  F  C  S  L  P  L  K  G  V  K  P  E  V  R  T  Y  N  I
1621   atgattggaggattatgtaagaaaggctcgctgcctgaagcggtcttgttgtttagaaagatgggagaggctggggttgcgccaagcagt
       M  I  G  G  L  C  K  K  G  S  L  P  E  A  V  L  L  F  R  K  M  G  E  A  G  V  A  P  S  S
1711   ggtacatacaacacactaatacgagctcatctcagaggtggtgacttaacaaaatcagctgaacttatcgaagaaatgaagaggtgtggg
       G  T  Y  N  T  L  I  R  A  H  L  R  G  G  D  L  T  K  S  A  E  L  I  E  E  M  K  R  C  G
1801   ttctctgcagatgcttcaaccataaagattgttatggatatgttattggatggtagaatgaagaaaagctttctggatatgctttcttag
       F  S  A  D  A  S  T  I  K  I  V  M  D  M  L  L  D  G  R  M  K  K  S  F  L  D  M  L  S  *
```

Supplementary Fig. S2. The putative promoter region and coding sequence of *Rfn* and its deduced amino acids from the restorer line H5.

```
1     atgttgtgtcggagattggtgcttgtgtctcgtatccccctgagtcctgttggtactcttgcaactcctttgctctcttttatcagatcc
      M  L  C  R  R  L  V  L  V  S  R  I  P  L  S  P  V  G  T  L  A  T  P  L  L  S  F  I  R  S
91    tcatgcgaacgaggctactctggtctcggcagcgatagaaatctctcatcttacaaagagagactgagaagtggtctggtcgatatcaag
      S  C  E  R  G  Y  S  G  L  G  S  D  R  N  L  S  S  Y  K  E  R  L  R  S  G  L  V  D  I  K
181   aagaaggatgctgtagctctgtttcagtccatgattaggtctcgtcctcttcctacggtcatggatttcaataaactgtttagtgcagta
      K  K  D  A  V  A  L  F  Q  S  M  I  R  S  R  P  L  P  T  V  M  D  F  N  K  L  F  S  A  V
271   gccagaacgaaacagtatgatctcgtgttggatctctgcaagcaaatggaactgcaagggattgcacatagcatttacacgctgagtatt
      A  R  T  K  Q  Y  D  L  V  L  D  L  C  K  Q  M  E  L  Q  G  I  A  H  S  I  Y  T  L  S  I
361   atgatcaattgcttctgccgtctgcgggaactcggttttgctttttctgtgatgggtaagatgttgaggcttgggtatgagcctgacaca
      M  I  N  C  F  C  R  L  R  E  L  G  F  A  F  S  V  M  G  K  M  L  R  L  G  Y  E  P  D  T
451   atcacattctcaactttgatcaacggtttatgtctggtgggtagagtttccgaagctgtggagttagttgatcgtatggtggaaatggag
      I  T  F  S  T  L  I  N  G  L  C  L  V  G  R  V  S  E  A  V  E  L  V  D  R  M  V  E  M  E
541   gttataccaaatctcatcacgctcaacactattgtcaatggactttgtctccaaggtgaagtgtctgaggcaatggctttgatcgatcga
      V  I  P  N  L  I  T  L  N  T  I  V  N  G  L  C  L  Q  G  E  V  S  E  A  M  A  L  I  D  R
631   atgatggataatggatgccaacccaatgaacgtacctatggtccggttttgaacagaatgtgcaagtcaggtaacactgccttggccttg
      M  M  D  N  G  C  Q  P  N  E  R  T  Y  G  P  V  L  N  R  M  C  K  S  G  N  T  A  L  A  L
721   gatctgctcagaaagatggaacacagaaagatcaagctcgatgcagtcacatacaatttcatcattgacagtctttgcaaagatgggagc
      D  L  L  R  K  M  E  H  R  K  I  K  L  D  A  V  T  Y  N  F  I  I  D  S  L  C  K  D  G  S
811   ctcgaagatgcactcagccttttcaatgaaatggaaaccaaaggtatcaaaccaaatgtctttacctacaactctctcattagaggcttc
      L  E  D  A  L  S  L  F  N  E  M  E  T  K  G  I  K  P  N  V  F  T  Y  N  S  L  I  R  G  F
901   tgtagtgctggaagatgggatgatggtgcaccgttgctgagggatatgatcacaaggggaatcacccccaccgtcatcactttcaattct
      C  S  A  G  R  W  D  D  G  A  P  L  L  R  D  M  I  T  R  G  I  T  P  T  V  I  T  F  N  S
991   ttgattgatagttttgtgaaagtgggaaagcttactgaggctcaagatttgtacaacgagatgatcacaagaggcacatatcctgatatc
      L  I  D  S  F  V  K  V  G  K  L  T  E  A  Q  D  L  Y  N  E  M  I  T  R  G  T  Y  P  D  I
1081  attacatataactctatgataaatgggctgtgcaatgaaaaacgcttagatgaagccaaccagatgctggatctgatggttagcaaggaa
      I  T  Y  N  S  M  I  N  G  L  C  N  E  K  R  L  D  E  A  N  Q  M  L  D  L  M  V  S  K  E
1171  tgcgatcctgatatcgtgacttataatacccttataaatggatactgtaaggctaaacgagttgatgaaggtatgagacatttccgcaaa
      C  D  P  D  I  V  T  Y  N  T  L  I  N  G  Y  C  K  A  K  R  V  D  E  G  M  R  H  F  R  K
1261  atgtctgtcaaaggagtggttgccaatacagtcacttataacactctcatccaagggttttgtcaatcaggaaaacttaatgttgccaaa
      M  S  V  K  G  V  V  A  N  T  V  T  Y  N  T  L  I  Q  G  F  C  Q  S  G  K  L  N  V  A  K
1351  gaactcttccaggagatggtctctcaaggtgttcatcctgatattataacctacaaaattttgctggatggattgtgtgacaatggagaa
      E  L  F  Q  E  M  V  S  Q  G  V  H  P  D  I  I  T  Y  K  I  L  L  D  G  L  C  D  N  G  E
1441  gtagaagaggctttgggaatacttgatcaaatgcacaagagtaacatggaacttgatattggtttatataatatcatcattcacgggatg
      V  E  E  A  L  G  I  L  D  Q  M  H  K  S  N  M  E  L  D  I  G  L  Y  N  I  I  I  H  G  M
1531  tgcaatgcaaataaggtcgatgatgcttggagtttgttctgtagcctacgttcgaaaggagtgaaaccagacgtcaagacatatactaca
      C  N  A  N  K  V  D  D  A  W  S  L  F  C  S  L  R  S  K  G  V  K  P  D  V  K  T  Y  T  T
1621  atgattggaggattgtgtaagaaaggatcgctgtctgaagcgggcatgttatgtaagaagatggaagaggatgggattgcgccaaatgat
      M  I  G  G  L  C  K  K  G  S  L  S  E  A  G  M  L  C  K  K  M  E  E  D  G  I  A  P  N  D
1711  tgtacatacaacactcttatcagggcacatctccgagatggtgacttaacaaaatcagcaaaacttatcgaagaaatgaagaggtgtggg
      C  T  Y  N  T  L  I  R  A  H  L  R  D  G  D  L  T  K  S  A  K  L  I  E  E  M  K  R  C  G
1801  ttctctgcagatgcttcaaccataaagattgttatggatatgttatcggatggtagaatgaagaaaagctttctggatatgctttcttag
      F  S  A  D  A  S  T  I  K  I  V  M  D  M  L  S  D  G  R  M  K  K  S  F  L  D  M  L  S  *
```

Supplementary Fig. S3. Nucleotide sequence of recessive *rfn* allele from 181A and the deduced amino acids. The mutations are indicated by red.

```
RFL2        MVKL---MIRRLSSQVSKFVQPRLLETGTLRIALINCPNELSFCCERGFSAFSD-R-NLS    55
RFN         MLCR----------RLVLVSRIPLSPVGTLAT---PLLSFIRSSCERGYSGLGSDRNLSS    47
RFP         MVLRTQRWNRLTTLRLVHLR---STETGTLRNAAFFQSPYEFFFCVQGFSGLTSDRKMSS    57
            *                          ***              *  * *     *   *

RFL2        YRERLRSGLVDIKADDAIDLFRDMIHSRPLPTVIDFSRLFSAIAKTKQYDLVLALCKQME    115
RFN         YKERLRSGLVDIKKKDAVALFQSMIRSRPLPTVMDFNKLFSAVARTKQYDLVLDLCKQME    107
RFP         YKERLRSGLVDIKKDDAVALFQSMLRSRPLPTVIDFNRLFGLLARTKQYDLVLALCKQME    117
            * ***********  **  **  *  ******* **  **   * ******** ******

RFL2        LKGIAHNLYTLSIMINCFCRCRKLCLAFSAMGKIIKLGYEPNTITFSTLINGLCLEGRVS    175
RFN         LQGIAHSIYTLSIMINCFCRLQKLGFAFSVMGKMLKLGYEPDTITFSTLINGLCLVGRVS    167
RFP         LKGIAYDLYTLNIMINCFCRRRKLGFAFSAMGKIFKLGYEPNTVTFNTLLNGLCLEGRVF    177
            * ***   *** ********  **  *** ***  ****** * ** ** ***** ***

RFL2        EALELVDRMVEMGHKPDLITINTLVNGLCLSGKEAEAMLLIDKMVEYGCQPNAVTYGPVL    235
RFN         KAVELVDHMVDMKVIPNLIILNTIVNGLCLQDRLSEAMSLIHRMLANGCQPDAVTYGPVL    227
RFP         EAVELVDCMVLSQHVPDLITLNTIVNGLCLKDRVSEAVDLIARMMANGCQPNQFTYGPIL    237
             * **** **     * **  ** ******     **  **  *   ****   **** *

RFL2        NVMCKSGQTALAMELLRKMEERNIKLDAVKYSIIIDGLCKHGSLDNAFNLFNEMEMKGIT    295
RFN         NRMCKSGNTASALDLLGKMELRKIKPQVVTYNIIIDSLCKDGSLEDALCLFNEMETKGIK    287
RFP         NRMCKSGNTASALDLLRKMEHRKIKPHVVTYTIIIDNLCKDGRLDDALSFFSEMETKGIK    297
            * ***** ** *  ** *** * **   * * **** *** * *  *   * *** ***

RFL2        TNIITYNILIGGFCNAGRWDDGAKLLRDMIKRKINPNVVTFSVLIDSFVKEGKLREAEEL    355
RFN         ANVITYTSLIGGFCSAGRWDDGAQLLRDMITRGVTPNVVTFNALIDSFVKEGQLTEAKKL    347
RFP         ANVFTYNSLIGSFCSFGRWDDGAQLLRDMITRKITPNVVTFSALIDSLVKEGKLTEAKDL    357
             *  **  *** **  ******* ****** *   ******  **** **** * **  *

RFL2        HKEMIHRGIAPDTITYTSLIDGFCKENHLDKANQMVDLMVSKGCDPNIRTFNILINGYCK    415
RFN         YNEMITRGTDPNIITYNSLIYGMCMDNCLDEANQMLDMMVSKGFYPDIVTFNILINGYCK    407
RFP         YNEMITRGIEPNTITYNSLIYGLCNDKRLDEANQMMDLMVSKGCDPDIWTYNILINGFCK    417
              *** **  *  *** *** * *    ** **** * *****  * * * ****** **

RFL2        ANRIDDGLELFRKMSLRGVVADTVTYNTLIQGFCELGKLNVAKELFQEMVSRKVPPNIVT    475
RFN         AKQVDEGTRLFRNMCLRGVVADTVTYNTLIQGFCQSGKLNVAKELFQEMVFVGVPPSVVT    467
RFP         AKQVDDGMRLFRKMSLRGMIADTVTYSTLIQGFCQSRKLIVAKKVFQEMVSQGVHPGIMT    477
            *   * *  *** * ***  ****** *******   ** ***  *****   * *   *

RFL2        YKILLDGLCDNGESEKALEIFEKIEKSKMELDIGIYNIIIHGMCNASKVDDAWDLFCSLP    535
RFN         YSILLDGLCDNGELEKALEILDQMLKSKMELDIGIYSIIIHGMCNASKIDDAWDLFCSLP    527
RFP         YAILLDGLCDNGELEEALGILDQMHKCKMELDIGIYNIIIHGMCNANKVDDAWSLFCSLS    537
            * *********** * ** *     * ********* ********* * **** *****

RFL2        LKGVKPGVKTYNIMIGGLCKKGPLSEAELLFRKMEEDGHAPDGWTYNILIRAHLGDGDAT    595
RFN         LKGVKPEVRTYNIMIGGLCKKGSLPEAVLLFRKMGEAGVAPSSGTYNTLIRAHLRGGDLT    587
RFP         LKGVKRDIQSYNIMLSGLCKRSSLSEADALFRKMKEDGYEPDGCTYNTLIRAHLRGNDIT    597
            *****     ****  ****   * **  ***** * *  *   *** ******   * *

RFL2        KSVKLIEELKRCGFSVDASTIKMVIDMLSDGRLKKSFLDMLS-----------    637
RFN         KSAELIEEMKRCGFSADASTIKIVMDMLLDGRMKKSFLDMLS-----------    629
RFP         TSVQLIEEMKRCGFSSDASTVKIVMDMLSSGELDKSFLNMLSGPFGDKSSSLD    650
             *  **** ****** **** * * ***  *   **** ***
```

Supplementary Fig. S4. Alignment of the amino acids sequences of RFN, RFP and their homologous protein RFL2 in *Arabidopsis*. The PPR repeats are highlighted by red and blue. The predicted mitochondrial signals are underline.

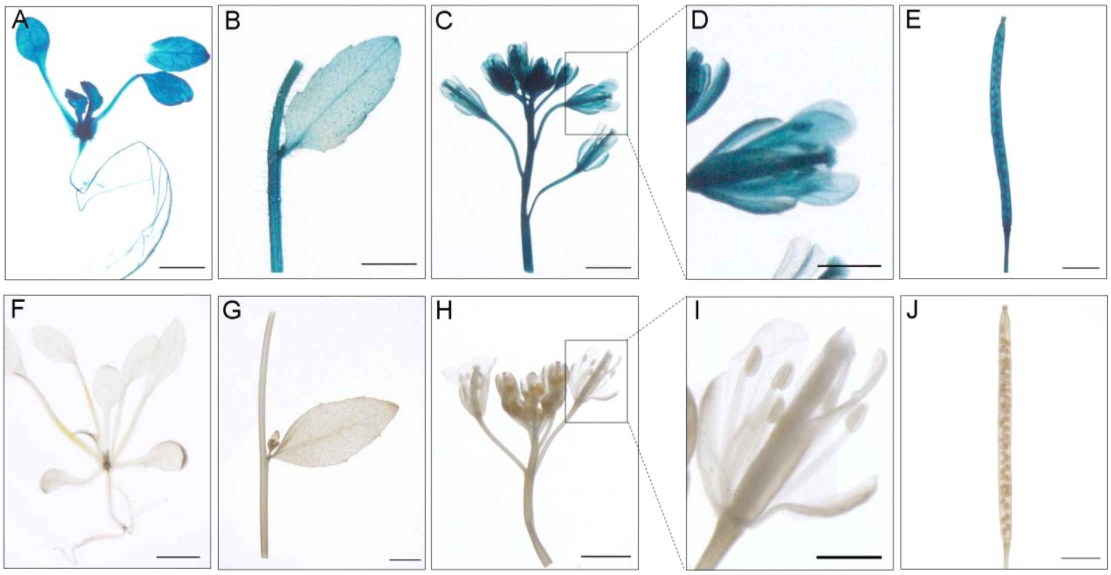

Supplementary Fig. S5. GUS assay from different tissues of Pro$_{Rfn}$: GUS introduced *Arabidopsis* plants. A, seedling after two weeks screened by hygromycin; B-F, staining of the stem and leaf (B), inflorescence (C), flower (D) and silique (E) from reproductive stage. F-J, staining of the corresponding tissues from wild-type plants. Scale bars: A-C, E-H, J, 2 mm; D, I, 500 μm.

Supplementary Table S1. The primers used in this study. Underlines indicate the restriction sites.

Prime name	Primer sequence (5'-3')	Purpose
BnSR02-F	CTCTTCTACGGTCAGTGGCA	Fine mapping
BnSR02-R	GAGATTGAGGATTGGGGTTG	
BnSR28-F	TTTGGAAAGAGCGGTTATGA	Fine mapping
BnSR28-R	ACATGCTCGATCGTGCTTAT	
BnSR33-F	GCTTGGTATCGTTCTCCGTC	Fine mapping
BnSR33-R	TCTGGCTTGTTACTCAAGCG	
BrSC64-F	CTGCTGCGAGTTACTTGAAGC	Fine mapping and BAC
BrSC64-R	AGGATTCTGCTTTTGTGGTTTC	screening
BrSC65-F	ATAGCAGAAGCAGCATTGAAAG	Fine mapping and BAC
BrSC65-R	GCCTGGAGTGAVAAGAAGACG	screening
BrIP77-F	TGACGTGAGGGCGATTCTTG	Fine mapping
BrIP77-R	TAATGGACCAGGCGGTGATG	
BrIP81-F	TCGCTCCACCAACAATAAAC	Fine mapping
BrIP81-R	TCAGTCCATGAGATCAAGACG	
ORF2-F	CGgaattcGTCCAACCACAATGGCTTTAAC	Binary construct of *Rfn*
ORF2-R	CGggatccATTGCTAAGGCGAATCCGGTTG	
Rfn-F	GTTCTTGCTGTAAAGCGTTGT	Transgenic plants
Rfn-R	GTTGCGTTTTCATTGCTTGTC	detection
RfnRT-F	CTTGCAGAGATCCAACACGAGAT	qRT-PCR of *Rfn* or *rfn*
RfnRT-R	CAAAACCGAGTTTCTGGAGGC	
Pro-F	CCttaattaaGTCCAACCACAATGGCTTTAAC	GUS-fusion
Pro-R	AggcgcgccTGTTTGTTTTTTTTTCTTCAAATCTAC	
Rfn-Xba-F	GCtctagaATGTTGTGTCGGAGATTGGT	GFP-fusion
Rfn-Xba-R	GCtctagaATTTCTATCGCTGCCGAGAC	
insitu-F	ATGTAAGAAAGGCTCGCTGC	RNA *in situ*
insitu-R	TAAGTCACCACCTCTGAGATGAG	hybridization

orf222-F	TGGTGGAAAAGATCGTACAAGT	RNA gel blot
orf222-R	AAGACCCGGAAGTGGTGATC	
orf139-F	CGATCCACAAACTGACGCAT	RNA gel blot
orf139-R	TTGTGCCGAGTCAAATCTGC	
BnActin-F	CCCTGGAATTGCTGACCGTA	qRT-PCR (control)
BnActin-R	TGGAAAGTGCTGAGGGATGC	

Supplementary Table S2. Fertility of T_1 progeny derived from five independent T_0 transgenic plants by selfing and test-cross.

No.	Source	Total plants	Fertile plants	Sterile plants	Expected ratio	χ^2 value [a]
T_1-04	selfing	61	47	14	3:1	0.05
T_1-20	selfing	96	70	26	3:1	0.13
T_1-21	selfing	132	125	7	15:1	0.07
T_1-29	selfing	122	111	11	15:1	1.16
T_1-46	selfing	99	91	8	15:1	0.30
T_1-04	test-cross	65	32	33	1:1	0
T_1-20	test-cross	88	51	37	1:1	1.92
T_1-21	test-cross	82	55	27	3:1	2.34
T_1-29	test-cross	78	60	18	3:1	0.07
T_1-46	test-cross	84	62	22	3:1	0.02

[a] χ^2 (0.05, 1) = 3.84
